# ^79/81^Br nuclear quadrupole resonance spectroscopic characterization of halogen bonds in supramolecular assemblies†
†Electronic supplementary information (ESI) available: ^13^C SSNMR spectra, powder X-ray diffractograms. See DOI: 10.1039/c8sc01094c


**DOI:** 10.1039/c8sc01094c

**Published:** 2018-04-30

**Authors:** P. Cerreia Vioglio, P. M. J. Szell, M. R. Chierotti, R. Gobetto, D. L. Bryce

**Affiliations:** a Department of Chemistry and NIS Centre , University of Torino , Via Pietro Giuria 7 , 10125 Torino , Italy; b Department of Chemistry and Biomolecular Sciences & Centre for Catalysis Research and Innovation , University of Ottawa , 10 Marie Curie Private , Ottawa , Ontario K1N 6N5 , Canada . Email: dbryce@uottawa.ca ; Fax: +1-613-562-5170 ; Tel: +1-613-562-5800 ext. 2018

## Abstract

Despite the applicability of solid-state NMR to study the halogen bond, the direct NMR detection of ^79/81^Br covalently bonded to carbon remains impractical due to extremely large spectral widths, even at ultra-high magnetic fields. In contrast, nuclear quadrupole resonance (NQR) offers comparatively sharp resonances. Here, we demonstrate the abilities of ^79/81^Br NQR to characterize the electronic changes in the C–Br···N halogen bonding motifs found in supramolecular assemblies constructed from 1,4-dibromotetrafluorobenzene and nitrogen-containing heterocycles. An increase in the bromine quadrupolar coupling constant is observed, which correlates linearly with the halogen bond distance (*d*_Br···N_). Notably, ^79/81^Br NQR is able to distinguish between two symmetry-independent halogen bonds in the same crystal structure. This approach offers a rapid and reliable indication for the occurrence of a halogen bond, with experimental times limited only by the observation of ^79/81^Br NQR resonances.

## Abstract 2

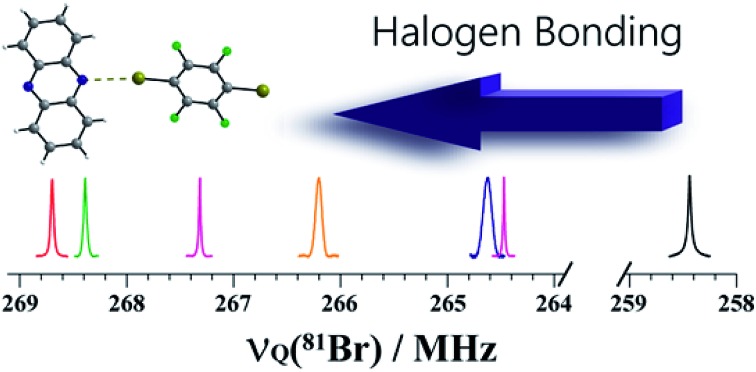
One- and two-dimensional bromine-79/81 NQR spectroscopy of halogen bond donors in a series of cocrystals shows changes in resonance frequency of up to 20 MHz and differentiates between crystallographically non-equivalent bromine sites.

## Introduction

The halogen bond (XB) consists of a non-covalent interaction between the area of lower electron density^1^ associated with a covalently bonded halogen atom, named the σ-hole,^2^,^3^ and a Lewis base. It is conveniently schematized as R–X···Y, where X is the halogen bond donor (strength: I > Br > Cl ≫ F),^4^ R is a group covalently bonded to X, and Y represents the electron-rich nucleophilic region of the halogen bond acceptor.^5^ With unique physicochemical properties such as directionality,^6^,^7^ strength,^8^,^9^ tunability,^10^,^11^ hydrophobicity,^12^ and selectivity,^13^,^14^ the halogen bond has become an important tool in modern supramolecular chemistry.^15^,^16^ Many promising applications are evident in medicinal chemistry,^17^–^19^ catalysis,^20^ and conductive materials,^21^ to name a few, and full literature reviews covering key advances in the field are available.^22^,^23^

Solid-state nuclear magnetic resonance (SSNMR) is a powerful tool to assess the occurrence of the halogen bond,^24^ to reliably determine phase purity,^25^ and to quantitatively relate local structural changes to geometrical features of the interaction.^26^,^27^ A key advantage of using SSNMR to characterize the halogen bond is the ability to non-destructively analyze samples in their powdered form, offering information on the chemical shift, quadrupolar coupling, dipolar coupling, and *J*-coupling.^25^,^27^–^32^ Notably, SSNMR experiments on ^13^C, ^15^N, ^31^P, or ^77^Se have been used at natural isotopic abundance to evaluate geometrical features of the halogen bond.^33^–^35^ Direct observation of the halogen bond donor has been limited to the study of ^35^Cl (nuclear electric quadrupole moment *Q*(^35^Cl) = –81.65(80) mb)^36^ covalently bonded to carbon,^31^ due to the broad spectral widths associated with the heavier halogens. This broadening arises as a consequence of the greater quadrupole moments of ^79/81^Br (*Q*(^79^Br) = 313(3) mb), ^81^Br (*Q*(^81^Br) = 262(3) mb), and especially ^127^I (*Q*(^127^I) = –696(12) mb),^36^ resulting in impractically broad solid-state NMR spectra. As the great majority of halogen-bonded compounds exhibit a halogen covalently bonded to a carbon atom,^23^ there have been various efforts towards the analysis of the ^13^C resonances for studying the halogen bond donor. However, ^13^C SSNMR spectroscopy of carbon covalently bonded to a quadrupolar halogen can be challenging due to the line shape distortion caused by residual dipolar coupling to the quadrupolar nucleus.^26^,^27^

As noted, ^79/81^Br and ^127^I both remain inaccessible by SSNMR when they are covalently bonded to carbon. Conversely, nuclear quadrupole resonance (NQR) offers advantages over SSNMR to directly characterize the XB; it enables the direct detection of the XB donor site and does not require an external magnetic field. For an exposition of the relative advantages and disadvantages of SSNMR and NQR, readers are referred to a recent Concepts article.^37^ The NQR frequencies for spin-3/2 nuclides, such as ^79^Br and ^81^Br, are a product of the quadrupolar coupling constant (*C*_Q_) and the asymmetry parameter (*η*), given by eqn (1):1
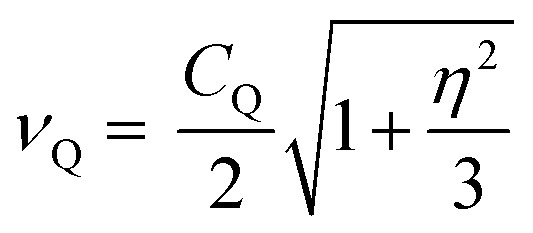

2
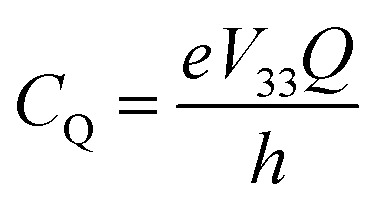

3
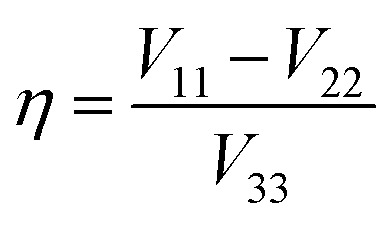
In eqn (2) and (3), *e* refers to the fundamental charge; *V*_11_, *V*_22_, and *V*_33_ refer to components of the electric field gradient tensor (|*V*_33_| ≥ |*V*_22_| ≥ |*V*_11_|); *Q* refers to the quadrupole moment of the nucleus; *h* refers to Planck's constant. In turn, the quadrupolar coupling parameters yield information on the electric field gradient (EFG) at the nucleus, providing information on electronic structure and bonding.^38^

**Fig. 1:**
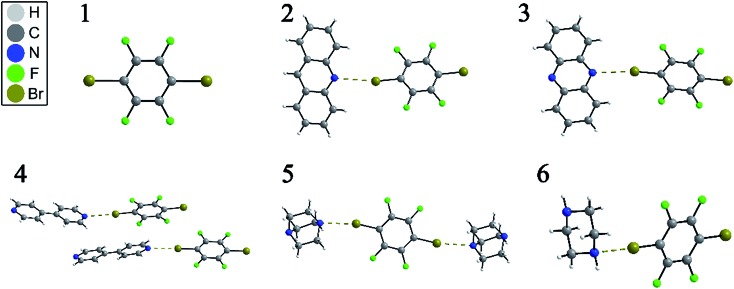
Depiction of the X-ray crystal structures of *p*-dibromotetrafluorobenzene (**1**) and its cocrystals (**2–6**), showing the C–Br···N halogen bond by dashed brown lines.

NQR has long played a role in characterizing charge-transfer complexes, but sparse information exists on the “halogen bond” as defined above.^39^–^42^ Consequently, the newest class of iconic halogen bond donors has not been thoroughly studied by NQR due in part to the fact that NMR has largely overtaken the field. Furthermore, clear and general relationships between the NQR frequencies and particular geometrical features have not yet been identified. Here, we report a systematic study of a series of prototypical C–Br···N XB motifs exhibiting different XB lengths and strengths (see Fig. 1) by ^79^Br and ^81^Br NQR spectroscopy. The geometrical features of the halogen bonds in each supramolecular assembly are summarized in Table 1. As the EFG at ^79^Br and ^81^Br are identical, the difference in the measured quadrupolar coupling constants for both isotopes is due to their different quadrupole moments (*Q*). Therefore, the ^79^Br and ^81^Br NQR frequencies should be related by a factor of ∼1.19 [*Q*(^79^Br)/*Q*(^81^Br)], providing a built-in verification of the experimental results.

**Table 1:** Geometrical parameters of the halogen bonds in compounds **1–6**, including the normalized distance parameter (*R*_XB_), the C–Br···N halogen bond angle (*θ*_C–Br···N_), and the Br···N halogen bond length (*d*_Br···N_)

Entry	Compound	CSD ref. 43	*R*_XB_ ^*a*^	*θ*_C–Br···N_ (°)	*d*_Br···N_ (Å)	Note
**1**	*p*-Dibromotetrafluorobenzene	ZZZAVJ ref. 44	—	—	—	
**2**	(Acridine)(**1**)	712 047 ref. 45	0.891^*b*^	172.13^*b*^	3.031^*b*^	
**3**	(Phenazine)(**1**)	712 045 ref. 45	0.878	172.59	2.985	
**4**	(4,4′-Bipyridine)(**1**)	199 297 ref. 46	0.846	177.21	2.878	Site 4A
			0.876	176.40	2.979	Site 4B
**5**	(1,4-Diazabicyclo[2.2.2]octane)(**1**)	649 676 ref. 47	0.851	167.69	2.894	Site 5A
			0.856	169.57	2.910	Site 5B
**6**	(Piperazine)(**1**)	649 675 ref. 47	0.847	177.72	2.881	

^*a*^The normalized distance parameter *R*_XB_ has been calculated as the ratio between the halogen bond length (*d*_Br···N_) and the sum of the van der Waals radii of Br and N.

^*b*^The X-ray crystal structure shows disorder on the position of the nitrogen, resulting in two possible halogen bond geometries; the reported values herein are the averages over the two disordered halogen bond sites.

## Results and discussion

**Fig. 2:**
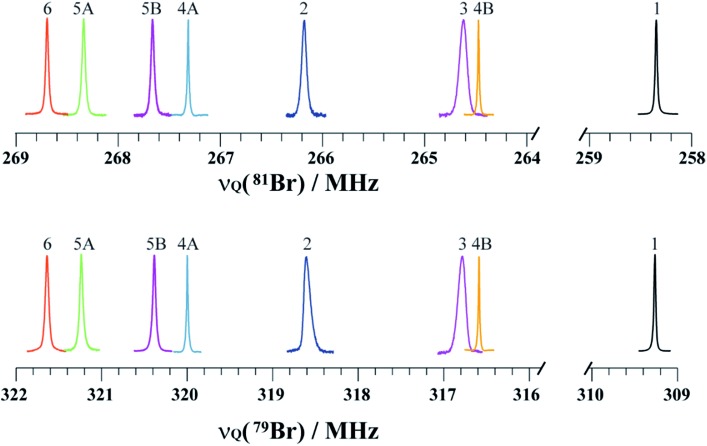
Pure ^81^Br and ^79^Br NQR resonances of compounds **1–6** depicted on a unified scale to emphasise the shift in the NQR resonance frequencies. The two crystallographically inequivalent bromine sites in compounds **4** and **5** are denoted **4A** & **4B** and **5A** & **5B**, respectively. Regions between 264 and 259 MHz (^81^Br) & 316 and 310 MHz (^79^Br) did not have any resonances, and were removed for clarity.

**Fig. 3:**
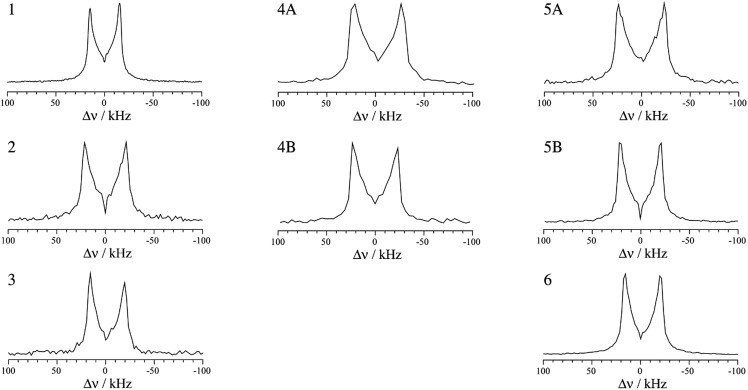
^81^Br nutation-echo NQR spectra of compounds **1–6**. The most intense *F*1 slices are shown. As the positive part is merely the antisymmetric image of the negative half, the differences in intensity between the positive and the negative half are likely due to RF inhomogeneity.

The structures of pure *p*-dibromotetrafluorobenzene (*p*-DBrTFB) and a series of cocrystals featuring C–Br···N halogen bonds are shown in Fig. 1. The NQR experiments, consisting of one-dimensional spectra and nutation data, are presented in Fig. 2 and 3, respectively. The data obtained from the ^81^Br and ^79^Br NQR experiments are summarized in Tables 2 and 3, respectively.

**Table 2:** Experimental and DFT-calculated ^81^Br NQR frequencies (*ν*_Q_), and quadrupolar parameters (*η*, *C*_Q_) of the halogen bonded compounds under investigation

Compounds	*ν*_Q_ exp. (MHz)	*ν*_Q_ calc.^*a*^ (MHz)	*η* exp.^*b*^	*η* calc.	*C*_Q_ exp. (MHz)	*C*_Q_ calc. (MHz)	FWHM (kHz)
**1**	258.34 ± 0.02	264.8	0.13 ± 0.04	0.114	515.23 ± 1.79	528.4	23
**2**	266.22 ± 0.04	268.9	0.15 ± 0.07	0.117	530.45 ± 3.72	536.6	66
**3**	264.64 ± 0.04	266.9	0.12 ± 0.09	0.109	528.01 ± 3.81	532.8	84
**4A**	267.32 ± 0.01	270.4	0.10 ± 0.08	0.117	533.75 ± 2.85	539.5	17
**4B**	264.47 ± 0.01	269.5	0.14 ± 0.06	0.125	527.22 ± 2.95	537.7	15
**5A**	267.66 ± 0.04	268.7	0.15 ± 0.05	0.120	533.32 ± 2.67	536.2	30
**5B**	268.33 ± 0.04	268.8	0.14 ± 0.06	0.119	535.02 ± 3.00	536.3	40
**6**	268.69 ± 0.02	269.2	0.16 ± 0.06	0.116	535.10 ± 3.43	537.1	26

^*a*^The calculated *ν*_Q_ frequencies were obtained using eqn (1) and the corresponding calculated *η* and *C*_Q_ values.

^*b*^Measured from the ^81^Br nutation NQR spectra.

**Table 3:** Experimental and DFT-calculated ^79^Br NQR frequencies (*ν*_Q_), and quadrupolar parameters (*η*, *C*_Q_) of the halogen bonded compounds under investigation

Compounds	*ν*_Q_ exp. (MHz)	*ν*_Q_ calc.^*a*^ (MHz)	*η* exp.^*b*^	*η* calc.	*C*_Q_ exp. (MHz)	*C*_Q_ calc. (MHz)	FWHM (kHz)
**1**	309.32 ± 0.02	316.9	0.13 ± 0.04	0.114	616.92 ± 2.14	632.4	22
**2**	318.61 ± 0.05	321.9	0.15 ± 0.07	0.117	634.84 ± 4.45	642.3	95
**3**	316.81 ± 0.04	319.5	0.12 ± 0.09	0.109	632.25 ± 4.55	637.7	98
**4A**	320.00 ± 0.02	323.6	0.10 ± 0.08	0.117	638.94 ± 3.41	645.8	17
**4B**	316.59 ± 0.02	322.6	0.14 ± 0.06	0.125	631.12 ± 3.54	643.5	16
**5A**	320.45 ± 0.03	321.7	0.15 ± 0.05	0.120	638.52 ± 3.19	641.8	43
**5B**	321.24 ± 0.04	321.7	0.14 ± 0.06	0.119	640.39 ± 3.59	641.9	45
**6**	321.65 ± 0.02	322.2	0.16 ± 0.06	0.116	640.57 ± 4.10	642.9	29

^*a*^The calculated *ν*_Q_ frequencies were obtained using eqn (1) and the corresponding calculated values of *η* and *C*_Q_.

^*b*^Measured from the ^81^Br nutation NQR spectra.

The search for the NQR resonances over the amplifier frequency range was the only time-determining step: once the resonance has been found, an excellent signal-to-noise ratio is achieved in about one minute on approximately 200 mg of sample. The signal frequency is characteristic of the local electronic environment at the bromine nucleus; hence, it provides direct information on the halogen bond. To the best of our knowledge, only a handful of data has been published in the literature regarding the characterization of the halogen bond by ^81^Br NQR.^48^ As shown in Fig. 2, the ^79^Br and ^81^Br NQR frequencies shift towards a higher frequency upon the formation of a halogen bond. As a general trend for the compounds studied herein, the shorter the halogen bond, the greater the shift (*vide infra*). This is consistent with previous ^81^Br NQR results on Br···N adducts.^48^

Notably, both ^79^Br and ^81^Br NQR provide clear differentiation between the two crystallographically inequivalent Br sites in compounds **4** and **5** (sites A and B), with the site assignments aided by DFT calculations. Importantly, in previous work on the C–Br···N motif, neither ^15^N SSNMR of the halogen bond acceptor nor ^13^C SSNMR of the halogen bond donor were able to discriminate two crystallographic sites.^26^ Additionally, due to the large quadrupole moments of ^79^Br and ^81^Br, the NQR frequencies are very sensitive to subtle changes in the crystallographic environment. For instance, a 3.41 ± 0.03 MHz difference is observed between the ^79^Br NQR frequencies of sites **4A** and **4B**, with a difference of 0.101 Å in *d*_Br···N_. In addition, comparing the two bromine sites in the X-ray crystal structure of **5** reveals a subtle difference of 0.016 Å in the *d*_Br···N_ between site **5A** and **5B**, while the value of θ_C–Br···N_ differs by merely 1.88°. Despite these very small geometrical differences, a clear and unambiguous difference of 0.79 ± 0.05 MHz is measured between the two ^79^Br NQR frequencies. In contrast, a ^13^C SSNMR analysis of these halogen-bonded compounds did not resolve the two crystallographically independent ^13^C sites due to residual dipolar coupling to both bromine isotopes (see ESI†).

For spin-3/2 nuclides such as ^79/81^Br, the pure one-dimensional NQR spectrum yields a single frequency which is related to the product of *C*_Q_ and *η* (see eqn (1)). In order to extract the individual EFG tensor components, several experimental methods have been proposed, such as Zeeman-perturbed NQR,^49^ nutation NQR,^50^,^51^ and level-crossing double resonance.^52^ Among these techniques, nutation NQR does not require a complex experimental setup, allowing for the determination of the quadrupolar asymmetry parameter in a straightforward manner. Implemented as a two-dimensional experiment, nutation NQR involves recording series of spectra where the pulse lengths are increased between each one-dimensional spectrum. This experiment allows for the observation of the orientation dependence of the quadrupolar interaction relative to the radiofrequency field. The result allows the measurement of *η*, which can then be used to determine the value of *C*_Q_.^50^

The highest intensity one-dimensional slices from the two-dimensional experimental ^81^Br nutation NQR spectra are shown in Fig. 3. Although a stronger RF field may improve the spectral line shapes, our home-built probe is limited to lower power levels. However, using the equation and method proposed for a spin-3/2 nucleus by Harbison,^50^,^51^ the experimental NQR line shapes have provided *η* values in agreement with the DFT calculated results (Tables 2 and 3). Confirmation bias was accounted for by measuring the maximum and minimum separation of the spectral singularities, thereby providing error limits on *η.*

**Fig. 4:**
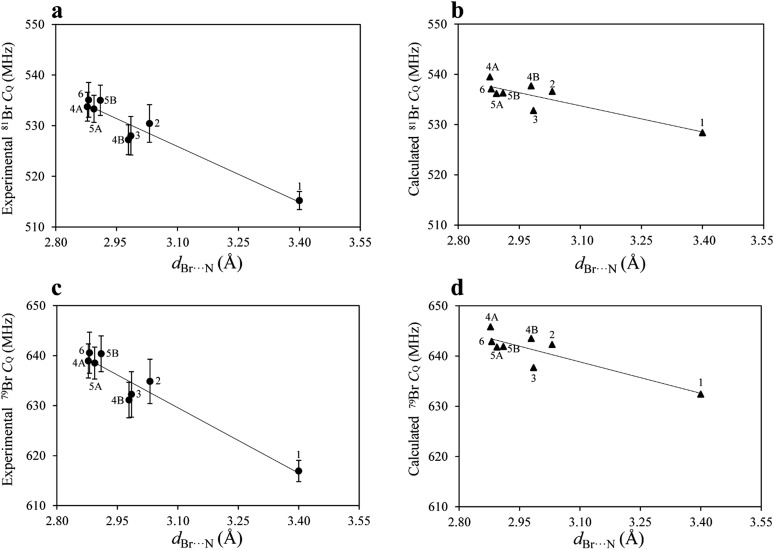
Plot of the experimental ^81^Br *C*_Q_ (a), calculated ^81^Br *C*_Q_ (b), experimental ^79^Br *C*_Q_ (c), and calculated ^79^Br *C*_Q_ (d) as a function of the Br···N halogen bond length. The lines of best fit and Pearson's correlation coefficients are: (a) *C*_Q_ = [–36.63 ± 4.41]*d*_Br···N_ + [639.45 ± 13.22], and *R*^2^ = 0.920; (b) *C*_Q_ = [–17.32 ± 4.03]*d*_Br···N_ + [587.44 ± 12.09], and *R*^2^ = 0.754; (c) *C*_Q_ = [–43.62 ± 5.14]*d*_Br···N_ + [764.81 ± 15.42], and *R*^2^ = 0.923; (d) *C*_Q_ = [–20.87 ± 4.84]*d*_Br···N_ + [703.54 ± 14.52], and *R*^2^ = 0.756.

After measuring *η* by ^81^Br nutation NQR, the values of *C*_Q_ for both isotopes were calculated using eqn (1), as both isotopes share the same *η* value. The *C*_Q_ values obtained for ^79^Br and ^81^Br are related by a ratio of 1.19, further confirming the experimental results. The DFT results are in good agreement with the experimental results, both in terms of *η* and *C*_Q_. Although the experimental and calculated *C*_Q_ data are close, the DFT results are systematically larger. While dispersion corrections were used in the calculations, the calculated *C*_Q_ values were not as strongly correlated to the halogen bond geometry as were the experimental data (Fig. 4). For instance, the experimental *C*_Q_(^79^Br) values show an average increase of about 18 MHz upon halogen bond formation, whereas the calculated results suggest an average increase of only 10 MHz.

Upon plotting both the experimental and calculated *C*_Q_ data as a function of the corresponding Br···N halogen bond distances, shown in Fig. 4, good linear correlations are observed. The origin of this effect can be mainly attributed to the interaction between the lone pair electrons from the nitrogen and the bromine atoms, which has historically been referred to as charge transfer. The increase in *C*_Q_ observed for ^79^Br and ^81^Br upon halogen bonding is in agreement with the results for chlorine, obtained from previous ^35^Cl SSNMR experiments.^31^ In the case of the bromine halogen bond, *C*_Q_ increases by 15 to 18 MHz, whereas in the case of chlorine halogen bonds, *C*_Q_ increases by 0.3 to 1.2 MHz.

The ^79^Br and ^81^Br NQR observables are remarkably sensitive to the halogen bonding environment, to such an extent that they provide better evidence for the occurrence of a halogen bond compared to the ^13^C and ^15^N SSNMR chemical shift of the carbon covalently bonded to iodine, or the nitrogen on the halogen bond acceptor. To the best of our knowledge, this is the first time that such a large effect has been observed for bromine atoms involved in halogen bonding.

## Conclusions

We have presented the first modern ^79/81^Br NQR study of halogen bonds, performed on a series of cocrystals based on 1,4-dibromotetrafluorobenzene and the C–Br···N motif. Through a combination of pure NQR and nutation NQR, we have measured both the quadrupolar coupling constant (*C*_Q_) and the asymmetry parameter (*η*). We demonstrate that this approach is sensitive to small changes in the halogen bond geometry, with the *C*_Q_ in good correlation with the XB length. The sensitivity of the *C*_Q_ values to the halogen bond geometry proved to be superior to ^13^C and ^15^N chemical shifts obtained by solid-state NMR, even allowing for the discrimination of several crystallographically inequivalent halogen bonds.

Although the time-determining factor of this technique was the search for the NQR resonances, the reported correlation can be used in future work to narrow the frequency range to be scanned. As NQR is performed in the absence of an applied magnetic field and does not require sophisticated equipment, it can be readily implemented as a tool to characterize the bromine halogen bond, in excellent complementarity with solid-state NMR and diffraction methods.

## Experimental

The starting materials were purchased from Sigma Aldrich and used without further purification. Solvents were purchased from Fisher Scientific and used as received. The synthesis of the cocrystal of 1,4-dibromotetrafluorobenzene (**1**, *p*-DBrTFB) with acridine (acd) to give **2** ((*p*-DBrTFB)(acd)), or phenazine (phz) to give **3** ((*p*-DBrTFB)(phz)), was performed as reported by Jones and collaborators.^45^ The synthesis of the cocrystal of **1** with 4,4′-bipyridine (bipy) to give **4** ((*p*-DBrTFB)(bipy)) was performed as reported by De Santis *et al.*^46^ The synthesis of the cocrystal of **1** with either 1,4-diazabicyclo[2.2.2]octane (dabco) to give **5** ((*p*-DBrTFB)(dabco)) or piperazine (pip) to give **6** ((*p*-DBrTFB)(pip)), was performed as reported by Cinčić *et al.*^47^ Powder X-ray diffraction and ^13^C CPMAS SSNMR were carried out to ensure phase purity (see the ESI†).

Pulsed ^79/81^Br NQR experiments were performed in the absence of an applied magnetic field using a Bruker Avance III 400 NMR spectrometer. A home-built probe was used, which consisted of a tuning capacitor, a matching capacitor, and a solenoid. All samples were ground and packed in 4 mm o.d. glass tubes prior to being placed inside the probe's RF coil for NQR analysis. Each spectrum was acquired using a Hahn–Echo pulse sequence (π/2–τ–π–τ–acquire), with a 3 μs π/2 pulse and 6 μs π pulse. A total of 256 or 1024 transients were acquired depending on the signal intensity, with a recycle delay of 0.5 s. In order to search for the NQR frequencies, the applied RF was incremented in steps of 150 kHz. The experimental spectra were fit using QUEST.^53^ Nutation NQR spectra were recorded as per ref. 50. DC correction was applied to each nutation spectrum.

Density functional theory (DFT) calculations were performed using the Amsterdam Density Functional (ADF) software^54^ with the metaGGA TPSS^55^ functional and the TZ2P basis set implemented in ADF. Dispersion forces were accounted for using Grimme3 BJDAMP.^56^ Scalar and spin–orbit relativistic effects were accounted for using ZORA^57^ as implemented in the ADF software.

## Conflicts of interest

There are no conflicts to declare.

## Supplementary Material

Supplementary information
