# Supplementary material for: 79/81Br nuclear quadrupole resonance spectroscopic characterization of halogen bonds in supramolecular assemblies
Source: Chem Sci. 2018 Apr 30;9(20):4555–61. doi: 10.1039/c8sc01094c (PMC5969492; doi:10.1039/c8sc01094c)
Supplement: Supplementary file 1 [file SC-009-C8SC01094C-s001.pdf]

Electronic Supplementary Material (ESI) for Chemical Science.
This journal is © The Royal Society of Chemistry 2018

$^{79/81}$Br Nuclear Quadrupole Resonance Spectroscopic Characterization of Halogen Bonds in Supramolecular Assemblies

P. Cerreia Vioglio,[a,†] P. M. J. Szell,[b] M. R. Chierotti,[a] R. Gobetto[a] and D. L. Bryce*,[b]

[a] Department of Chemistry and NIS Centre, University of Torino, Via Pietro Giuria 7, 10125 Torino, Italy.

[b] Department of Chemistry and Biomolecular Sciences & Centre for Catalysis Research and Innovation. University of Ottawa, 10 Marie Curie Private, Ottawa, Ontario K1N 6N5, Canada.

* Tel: +1-613-562-5800 ext.2018; Fax: +1-613-562-5170. Email: dbryce@uottawa.ca

† Present address: Aix-Marseille Université, CNRS, ICR (UMR 7273), 13397 Marseille cedex 20 (France).

List of Figures

Figure S1. Experimental ^{13}C solid-state NMR spectrum of **2** (p-DBrTFB)(acridine) 3
Figure S2. Experimental ^{13}C solid-state NMR spectrum of **3** (p-DBrTFB)(phenazine) 3
Figure S3. Experimental ^{13}C solid-state NMR spectrum of **4** (p-DBrTFB)(bipy) 4
Figure S4. Experimental ^{13}C solid-state NMR spectrum of **5** (p-DBrTFB)(dabco).................................... 4
Figure S5. Experimental ^{13}C solid-state NMR spectrum of **6** (p-DBrTFB)(pip) 5
Figure S6. Experimental powder X-ray diffraction of sample **1** 6
Figure S7. Experimental powder X-ray diffraction of sample **2** with the calculated pattern overlaid......... 6
Figure S8. Experimental powder X-ray diffraction of sample **3** with the calculated pattern overlaid......... 7
Figure S9. Experimental powder X-ray diffraction of sample **4** with the calculated pattern overlaid......... 7
Figure S10. Experimental powder X-ray diffraction of sample **5** with the calculated pattern overlaid....... 8
Figure S11. Experimental powder X-ray diffraction of sample **6** with the calculated pattern overlaid....... 8

$^{1}H \rightarrow ^{13}C$ Cross-Polarization Magic-Angle Spinning Solid-State NMR Spectra. B_0 = 9.4 T.

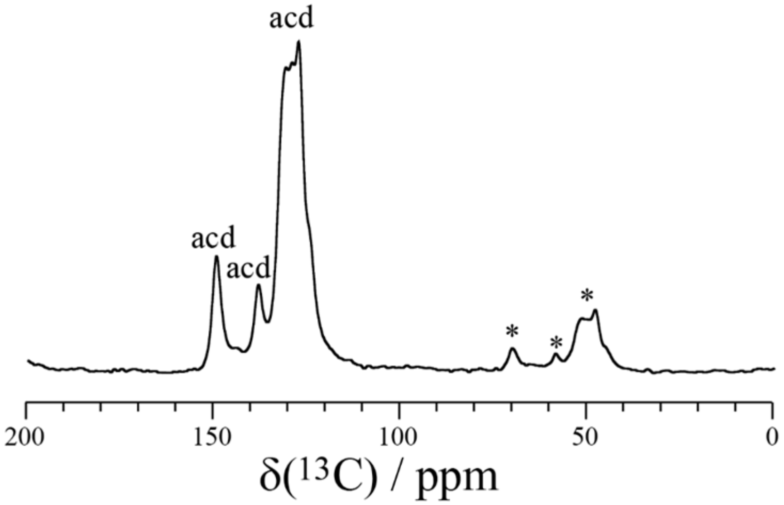

Figure S1. Experimental ^{13}C solid-state NMR spectrum of **2** (*p*-DBrTFB)(acridine). The "acd" labels denote the resonances assigned to acridine ($\delta(^{13}C)$ = 149.0 ± 0.3 ppm, 137.6 ± 0.3 ppm, 130.4 ± 0.5 ppm to 126.9 ± 0.4 ppm, left to right). The asterisks denote spinning sidebands.

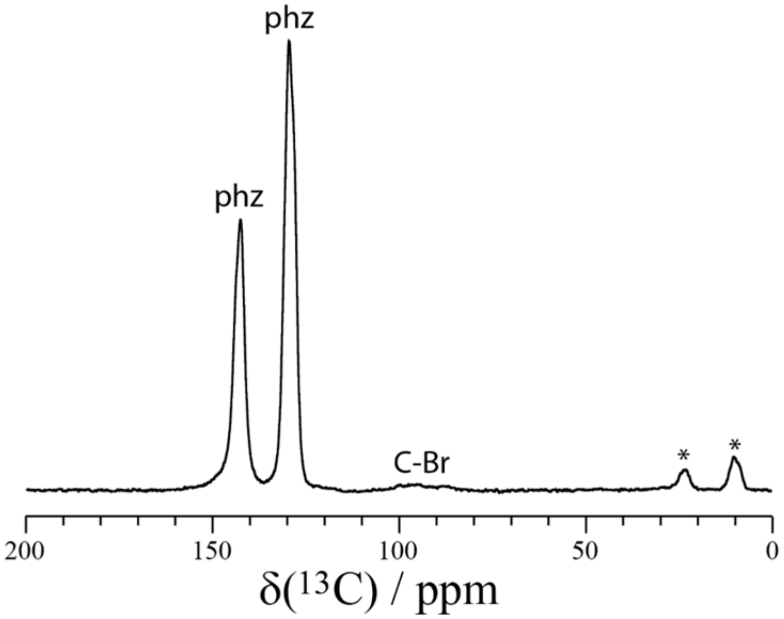

Figure S2. Experimental ^{13}C solid-state NMR spectrum of **3** (*p*-DBrTFB)(phenazine). The "phz" labels denote the resonances assigned to phenazine ($\delta(^{13}C)$ = 142.8 ± 0.6 ppm, 129.8 ± 0.7 ppm, left to right), whereas the "C-Br" label denotes the resonance assigned to the carbon covalently bonded to bromine ($\delta(^{13}C)$ = 100.8 ± 0.8 ppm to 85.8 ± 1.7 ppm). The asterisks denote spinning sidebands.

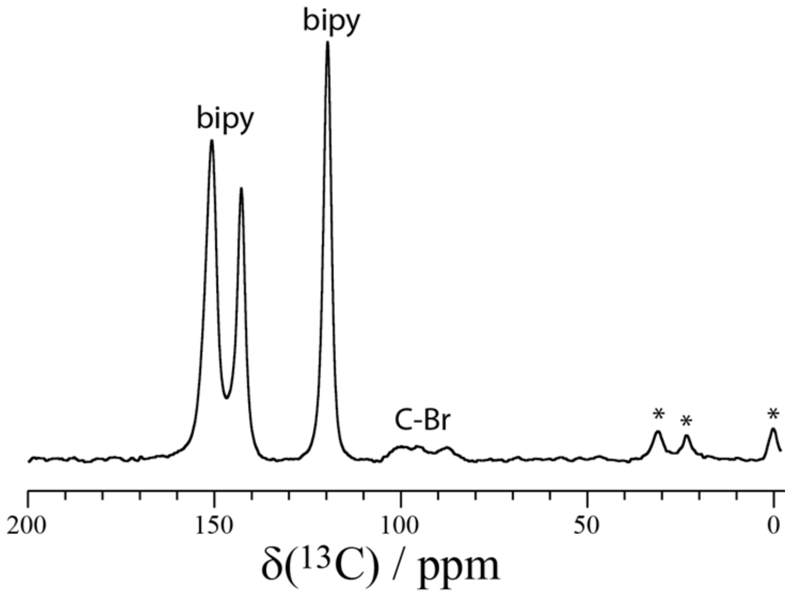

Figure S3. Experimental ^{13}C solid-state NMR spectrum of **4** (*p*-DBrTFB)(bipy). The "bipy" labels denote the resonances assigned to phenazine (δ(^{13}C) = 150.9 ± 0.4 ppm, 142.9 ± 0.4 ppm, 119.88 ± 0.2 ppm, left to right), whereas the "C-Br" label denotes the resonance assigned to the carbon covalently bonded to bromine (δ(^{13}C) = 103.3 ± 1.6 ppm to 85.3 ± 1.3 ppm). The asterisks denote spinning sidebands.

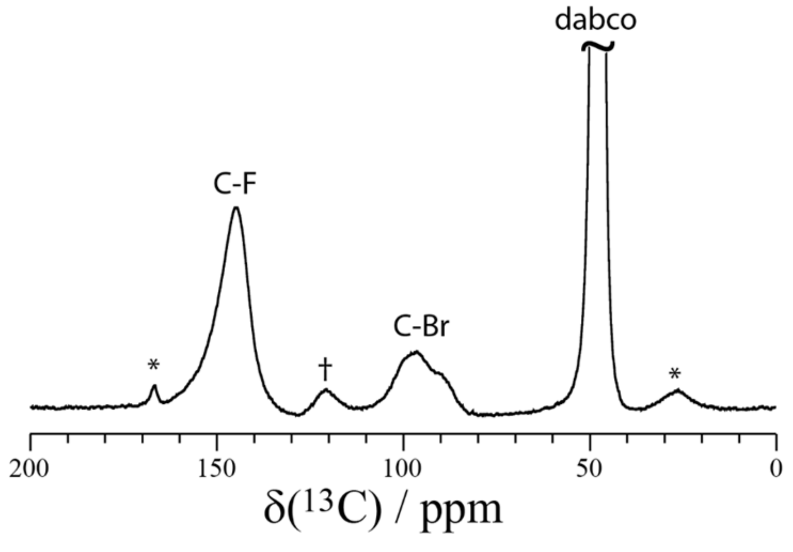

Figure S4. Experimental ^{13}C solid-state NMR spectrum of **5** (*p*-DBrTFB)(dabco). The "dabco" label denotes the resonance assigned to dabco (δ(^{13}C) = 47.6 ± 0.1 ppm), whereas the "C-Br" label denotes the resonance assigned to the carbon covalently bonded to bromine (δ(^{13}C) = 102.6 ± 1.3 ppm to 85.3 ± 2.1 ppm), and the "C-F" symbol denotes the carbon covalently bonded to fluorine (δ(^{13}C) = 102.6 ± 1.3 ppm). The asterisks denote spinning sidebands. The dagger indicates a trace impurity.

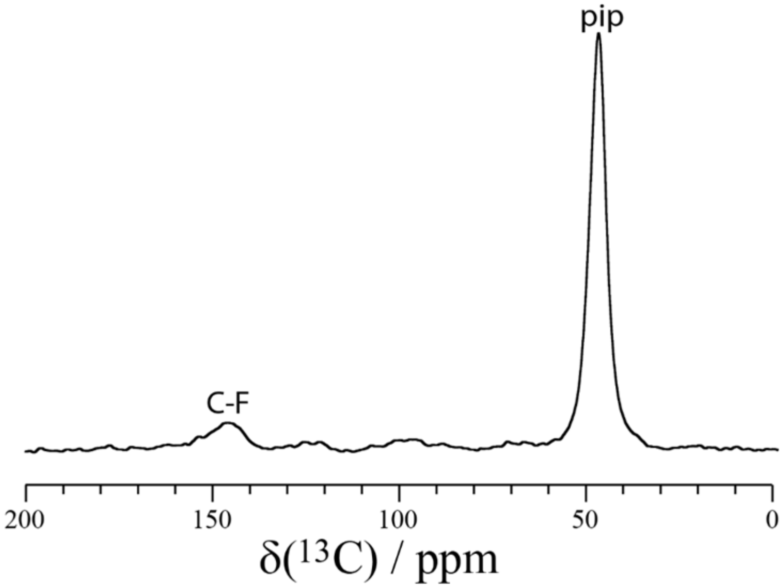

Figure S5. Experimental ^{13}C solid-state NMR spectrum of **6** (*p*-DBrTFB)(pip). The “pip” label denotes the resonance assigned to piperazine ($\delta(^{13}C)$ = 46.5 ± 0.4 ppm), whereas the “C-F” label denotes the resonance assigned the carbon covalently bonded to fluorine ($\delta(^{13}C)$ = 145.9 ± 2.3 ppm). The asterisks denote spinning sidebands.

Powder X-ray Diffraction

Powder X-ray diffractograms were acquired using a Rigaku Ultima IV Diffractometer operating at room temperature (298 ± 1 K) with a copper source and a diffracted beam monochromator.

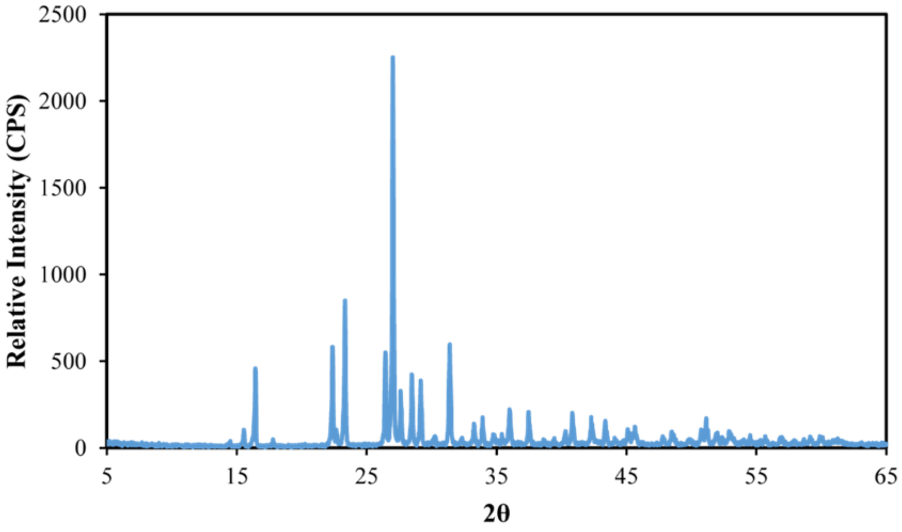

Figure S6. Experimental powder X-ray diffraction of sample **1**.

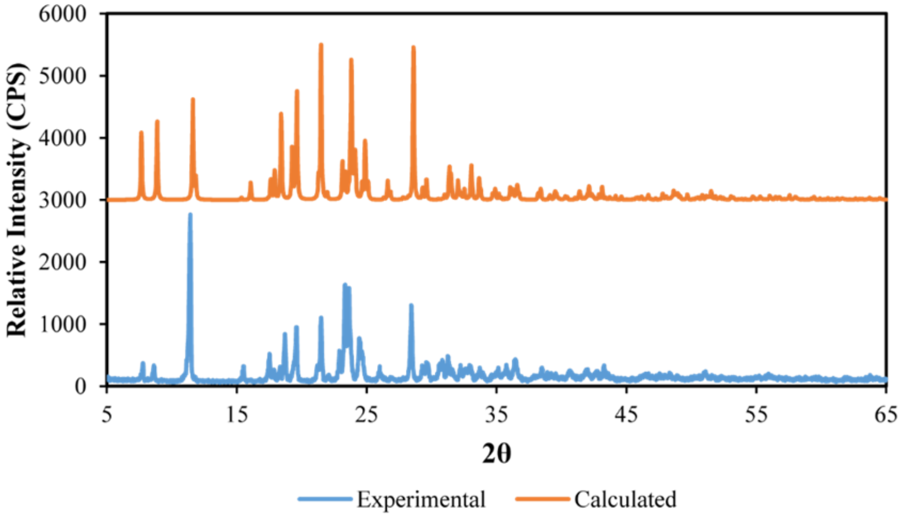

Figure S7. Experimental powder X-ray diffraction of sample **2** with the calculated pattern overlaid.

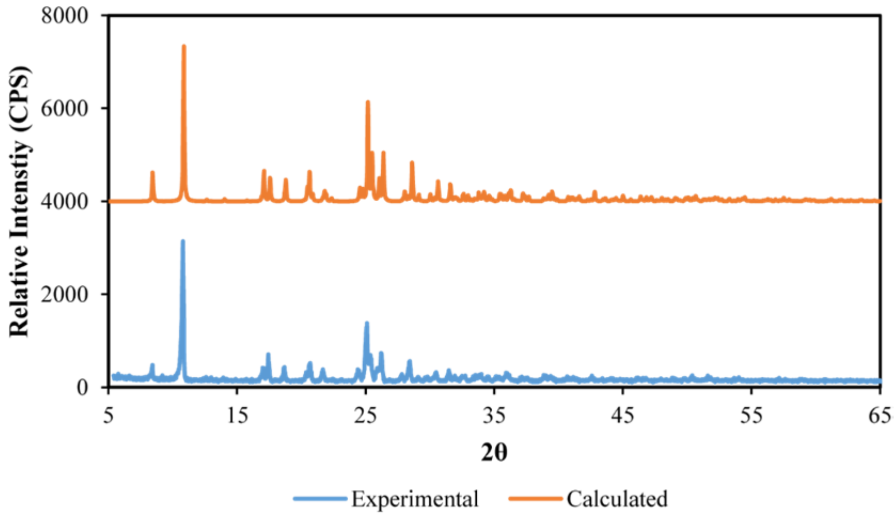

Figure S8. Experimental powder X-ray diffraction of sample **3** with the calculated pattern overlaid.

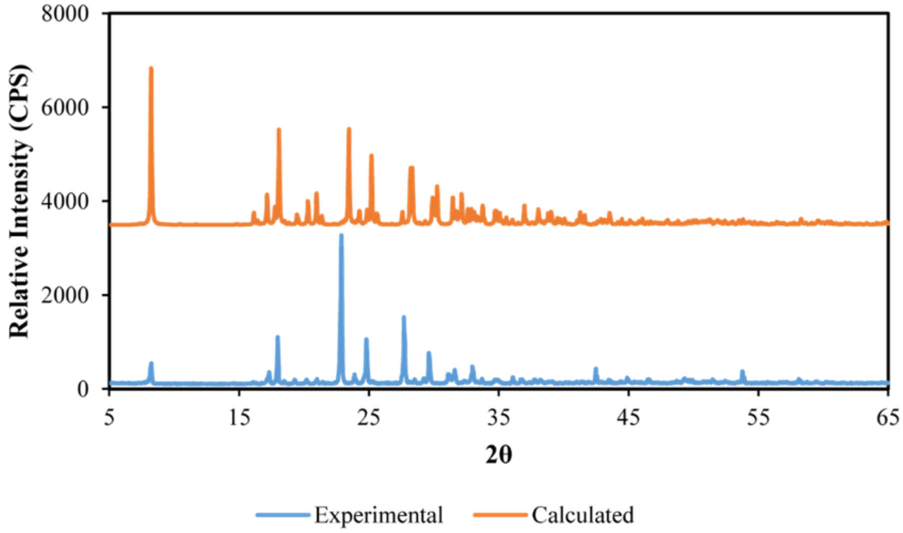

Figure S9. Experimental powder X-ray diffraction of sample **4** with the calculated pattern overlaid.

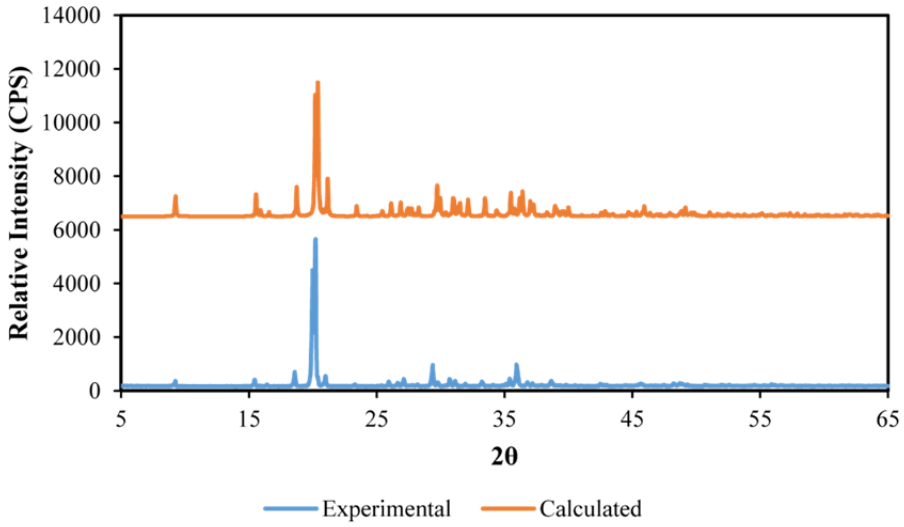

Figure S10. Experimental powder X-ray diffraction of sample **5** with the calculated pattern overlaid.

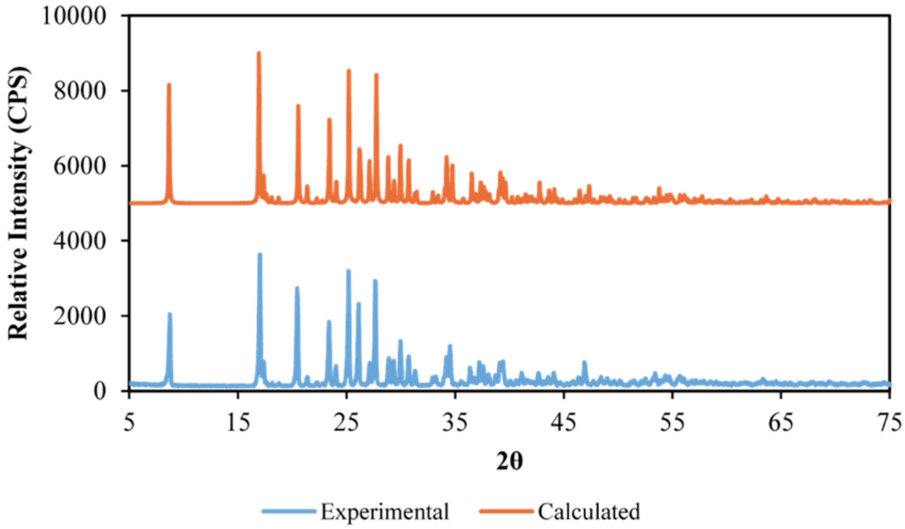

Figure S11. Experimental powder X-ray diffraction of sample **6** with the calculated pattern overlaid.
